# Клиническое наблюдение пациента с сочетанной эндокринной патологией: врожденной дисфункцией коры надпочечников и синдромом Клайнфельтера

**DOI:** 10.14341/probl13298

**Published:** 2024-03-11

**Authors:** Н. И. Волкова, И. Ю. Давиденко, Д. П. Ставицкая, Е. В. Кудинова

**Affiliations:** Ростовский государственный медицинский университет; Ростовский государственный медицинский университет; Ростовский государственный медицинский университет; Ростовский государственный медицинский университет

**Keywords:** врожденная дисфункция коры надпочечников, синдром Клайнфельтера, гиперандрогения, тестостерон

## Abstract

Врожденная дисфункция коры надпочечников (ВДКН) — это группа аутосомно-рецессивных заболеваний, характеризующихся дефектом одного из ферментов или транспортных белков, принимающих участие в синтезе кортизола в коре надпочечников [1]. Нарушение синтеза кортизола вследствие недостаточности фермента 21-гидроксилазы ведет к тому, что по механизму отрицательной обратной связи избыток АКТГ стимулирует корковый слой надпочечников, что вызывает ее гиперплазию. Но вместо кортизола надпочечники производят избыток предшественников половых гормонов, для синтеза которых не требуется 21-гидроксилирование. После секреции эти гормоны метаболизируются в активные андрогены — тестостерон и дигидротестостерон, и в меньшей степени — в эстрогены: эстрон и эстрадиол [2]. При вирильной форме отмечается только дефицит кортизола, что при отсутствии лечения проявляется мышечной слабостью, утомляемостью, потемнением кожных покровов на фоне симптомов гиперандрогении [1, 2]. Синдром Клайнфельтера (СК) — одно из наиболее частых хромосомных заболеваний, приводящее к развитию первичного гипогонадизма. Основное проявление синдрома Клайнфельтера — гиалиноз семявыносящих протоков и — как следствие — развитие инфертильности и гипогонадизма. Фенотипическая изменчивость, и особенно умеренные клинические проявления, часто приводят к задержке диагностики или недиагностированию [3]. Было подсчитано, что 50–75% мужчин с синдромом Клайнфельтера никогда не получают диагноз [4].

В данной статье представлено описание редкого клинического наблюдения: сочетание вирильной формы врожденной дисфункции коры надпочечников и синдрома Клайнфельтера, описаны особенности клинической картины заболеваний в период раннего детства, пубертата, среднего возраста.

Проявление двух этих заболеваний, при одном из которых наблюдается гиперандрогения, а при другом — гипогонадизм, у одного пациента обуславливает трудности диагностики и подбора терапии. Ошибки, которые могут быть допущены на различных этапах диагностики и лечения заболеваний у данного пациента, ведут к неблагоприятным последствиям и сказываются на качестве жизни больного. Представленный клинический случай демонстрирует сложности диагностики и необходимость повышения информированности врачей о данной клинической проблеме.

## Abstract (en)

Congenital adrenal hyperplasia (CAH) is a defect in one of the enzymes or transport proteins involved in the synthesis of cortisol in the adrenal cortex. Virile form of CAH characterized by cortisol deficiency and hyperandrogenism. Klinefelter syndrome is one of the most frequent chromosomal diseases leading to the development of primary hypogonadism. The manifestation of these two diseases could cause difficulties in diagnosis and medical treatment that lead to adverse consequences and affect the quality of life.

A 43-years-old patient consulted a physician complaining about the lack of erections for 4 years, breast enlargement. At the age of 3 years based on experienced growth of pubic hair, decreased level of 17-ketosteroids in the urine and genetic analysis diagnosis of CAH, virile form was suspected. Prednisone 5 mg daily was prescribed. At the age of 5, based on phenotypic features and karyotyping Klinefelter Syndrome (XXY) was diagnosed. At the age of 13, stimulating hormonal chorionic gonadotropin drug with only one course of 10 injections was prescribed. At the age of 18, the patient independently canceled the use of prednisone. Further, he did not receive medication therapy for CAH and Klinefelter syndrome. At the age of 42, adrenal CT revealed formation of the left adrenal gland. According to the results of the hormonal activity examination, high levels of aldosterone and renin were detected. A diagnosis of left adrenal aldosteroma was made and a left-sided adrenalectomy was performed. Histological examination diagnosis of aldosteroma did not confirmed. On physical examination, BMI 30 kg/m2, genoid type of obesity, right testicle isn`t palpated, left testicle is dense, reduced in size. Small penis size. Decreased level of total testosterone, normal level of SHBG, LH and FSH was revealed. Ultrasound of the scrotum organs revealed decrease in the size of the testicles and appendages, a volumetric formation of the right testicle. Thus, diagnosis of CAH, virile form and Klinefelter syndrome, primary hypogonadism, right-sided cryptorchidism was confirmed. Hydrocortisone 30 mg daily was prescribed. Hormone replacement therapy with testosterone preparations was not prescribed until surgical treatment of neoplasm of the right testicle will be performed. On the example of this clinical case, we have demonstrate a combination of two endocrine pathologies and serious mistakes were made in the management of this patient. The management of such patients requires a multidisciplinary approach, which will avoid mistakes and improve the prognosis and quality of life of these patients.

## АКТУАЛЬНОСТЬ

Врожденная дисфункция коры надпочечников (ВДКН) — это группа аутосомно-рецессивных заболеваний, характеризующихся дефектом одного из ферментов или транспортных белков, принимающих участие в синтезе кортизола в коре надпочечников. Заболеваемость классической формой ВДКН (дефицит 21-гидроксилазы) составляет от 1:14 000 до 1:18 000 живых новорожденных в мире [1]. Более 90% ВДКН вызвано дефицитом 21-гидроксилазы. Фенотип может варьировать от легкой (неклассической) до классических форм: вирильной и сольтеряющей. Сольтеряющая форма заболевания ассоциирована с дефицитом минералкортикоидов (альдостерона) и глюкокортикоидов. Классическая простая вирилизирующая форма заболевания может приводить к гонадотропиннезависимому преждевременному половому созреванию мужчин с уменьшением конечного роста. Крайне важно диагностировать данное заболевание и своевременно назначить заместительную гормональную терапию глюкокортикоидами, которая является основой лечения [1][2].

Синдром Клайнфельтера (СК) — одно из наиболее частых хромосомных заболеваний, приводящее к развитию первичного гипогонадизма. Распространенность синдрома Клайнфельтера составляет 1:500–1000 новорожденных мальчиков [5], возрастает до 3–4% среди бесплодных мужчин и до 10–12% у пациентов с азооспермией [3]. Частота постановки диагноза в детском возрасте чрезвычайно низка, и только 10% случаев выявляются до наступления половой зрелости, достигая 25% к взрослому возрасту. За последние десятилетия появилось много новой информации о долгосрочных последствиях синдрома, от которых страдают эти мужчины. СК связан не только с нарушением функции яичек и увеличением роста, он оказывает влияние на когнитивные функции, поведение и психические расстройства, состав тела и чувствительность к инсулину, формирование костей и риск переломов, а также негативно влияет на общую заболеваемость и смертность [6]. Пациентов с диагнозом «СК» необходимо наблюдать на протяжении всей жизни и лечить тестостероном в случае гипогонадизма.

Наличие у одного пациента синдрома Клайнфельтера с ВДКН из-за дефицита фермента 21-гидроксилазы представляет собой уникальный диагностический парадокс, когда признаки дефицита гонадных андрогенов маскируются одновременным избытком надпочечниковых андрогенов. Одновременное возникновение ВДКН и синдрома Клайнфельтера встречается крайне редко. В современной литературе описано не более шести подобных случаев. Так, у одного из пациентов был выявлен дефицит 3b-гидроксистероиддегидрогеназы [7], а у остальных верифицирован дефицит 21-гидроксидазы [8][9][10][11][12]. Интерес к описанию этого случая заключается в исключительности одновременного возникновения двух этих состояний. Это уникальное описание сосуществования двух заболеваний, первое из которых вызывает избыток, а второе — дефицит андрогенов. Этим же обусловлены трудности подбора терапии, ведь дефицит андрогенов, обусловленный тестикулярной недостаточностью, до определенного времени может компенсироваться избытком андрогенов надпочечником, более того, могут быть признаки избытка андрогенов, в частности преждевременное половое созревание. Тогда возникают вопросы: как сохранить баланс? Необходимо бороться с избытком андрогенов или же компенсировать их дефицит?

Данный клинический случай иллюстрирует, как взаимодействие между двумя расстройствами с противоположными эффектами на уровне андрогенов может создавать диагностические и терапевтические проблемы.

## ОПИСАНИЕ СЛУЧАЯ

Пациент И. 43 лет обратился в отделение эндокринологии с жалобами на отсутствие эрекций в течение четырех лет, увеличение молочных желез. Из анамнеза стало известно, что у пациента в возрасте 2 лет и 8 месяцев началось оволосение лобка, он был обследован, и на основании повышения уровня 17-кетостероидов в моче был заподозрен диагноз «Врожденная дисфункция коры надпочечников, вирильная форма». Диагноз был подтвержден путем проведения генетического анализа. В связи с этим пациенту был назначен преднизолон в дозе 5 мг в сутки. Со слов матери, пациент препарат принимал регулярно.

В возрасте 5 лет ввиду фенотипических особенностей (высокий рост, непропорционально длинные ноги, правосторонний крипторхизм), у пациента заподозрили хромосомное заболевание, было проведено кариотипирование, на основании чего пациенту был установлен диагноз «Синдром Клайнфельтера (ХХУ)». Рекомендовано динамическое наблюдение, низведение неопустившегося правого яичка не проводилось.

Пациенту ежегодно определяли уровень 17-кетостероидов в моче для контроля эффективности терапии преднизолоном, результаты были в пределах нормы.

В 13 лет при наступлении пубертата пациенту была назначена стимулирующая гормональная терапия препаратом ХГЧ курсом 10 инъекций. В дальнейшем данную терапию не получал, проводилось динамическое наблюдение. В возрасте 15 и 16 лет неоднократно зафиксировано снижение уровня общего тестостерона при высоконормальных ЛГ и ФСГ, однако гормональная терапия не назначалась.

В 18 лет пациент самостоятельно отменил прием преднизолона, поскольку посчитал дальнейший прием препарата нецелесообразным.

В последующие годы медикаментозную терапию по поводу ВДКН и синдрома Клайнфельтера не получал, к эндокринологу не обращался.

Пациент находится в браке, детей не имеет.

В возрасте 37 лет появились жалобы на снижение либидо, пациент обратился к эндокринологу, назначено гормональное обследование, по результатам которого выявлено снижение общего тестостерона до 5,7 нмоль/л (8,3–30 нмоль/л), повышение ЛГ до 16,6 мМЕ/мл (0,8–7,6 мМЕ/мл), повышение ФСГ до 32,7 мМЕ/мл (0,7–11,1 мМЕ/мл). В связи с этим рекомендовано начать заместительную гормональную терапию препаратами тестостерона в/м, однако пациент не соблюдал рекомендации. Наблюдалось прогрессирование эректильной дисфункции, что в итоге привело к полной утрате потенции в 39 лет.

В 42 года при плановом обследовании на КТ органов брюшной полости и забрюшинного пространства выявлено образование левого надпочечника размерами 87*74*84 мм. Для исключения гормональной активности образования надпочечника проведено лабораторное обследование: анализ крови на кортизол на фоне приема 1 мг дексаметазона, определение метанефрина и норметанефрина суточной мочи и анализ крови на альдостерон и ренин. По результатам обследования выявлен высокий уровень альдостерона 522 пг/мл (0–199 пг/мл) и ренина 82,8 мкМЕ/мл (4,4–46,1 меМЕ/мл). Несмотря на высокий уровень ренина и альдостерона состояние ошибочно расценено как альдостерома, и проведена левосторонняя адреналэктомия. Микроскопическое описание опухоли: в ткани надпочечника опухоль солидно-трабекулярного строения, состоящая из округлых клеток, схожих с клетками коры надпочечников, с эозинофильной зернистой цитоплазмой и округлыми относительно мономерными ядрами, с единичными фигурами митозов, наличием в цитоплазме части клеток пигмента коричневого цвета. Среди опухолевой ткани отмечаются липоматозные и миелолипоматозные очаги, кровоизлияния, очаги коагуляционного некроза. Признаков инвазивного роста, клеточной атипии не обнаружено. Пациент был выписан с диагнозом: «Аденома левого надпочечника, состояние после лапароскопической адреналэктомии слева».

Спустя год после операции пациенту было проведено повторное КТ забрюшинного пространства, по результатам которого отмечалась гиперплазия правого надпочечника. Был заподозрен рецидив альдостеромы, повторно проведено определение альдостерона крови, и получен повышенный уровень альдостерона 366 пг/мл (0–199 пг/мл).

Пациент был госпитализирован в эндокринологическое отделение для подтверждения диагноза и определения тактики лечения.

## Результаты физикального, лабораторного и инструментального исследований

Данные осмотра: рост — 178 см, вес — 97 кг, ИМТ — 30 кг/м², ОТ — 109, телосложение гиперстеническое. АД —120/80 мм рт.ст. без гипотензивной терапии. Нарушение пропорций тела (длинные конечности, узкая грудная клетка). Геноидный (женский) тип ожирения. Слаборазвитая скелетная мускулатура. При пальпации правое яичко не пальпируется, левое яичко плотное, уменьшено в размере. Малый размер полового члена (4 см).

В отделении пациенту проведено лабораторное обследование, по результатам которого выявлено повышение уровня 17 ОН прогестерона до 145 нг/мл (0,5–2,1 нг/мл), уровень кортизола — 4,4 мкг/дл (3,17–19,4 нг/мл), уровень АКТГ — 67,19 пг/мл (7,2–63,3 пг/мл), снижение уровня общего тестостерона до 13,3 нмоль/л (8,3–30 нмоль/л), нормальный уровень ГСПГ — 35,5 нмоль/л (16,2–68,5 нмоль/л), произведен расчет уровня свободного тестостерона, по результатам которого выявлено снижение свободного тестостерона — 243 пмоль/л, высокий уровень ЛГ — 8,0 мМЕ/мл (0,8–7,6 мМЕ/мл) и ФСГ — 8,5 мМЕ/мл (0,7–11,1 мМЕ/мл), нормальный уровень пролактина — 267,1 мМЕ/л (57–600 мМЕ/л), тиреоидные гормоны в пределах нормы (ТТГ — 2,4 мМЕ/л, свТ4 — 18,7 пмоль/л).

Проведены инструментальные обследования. По результатам УЗИ органов мошонки выявлен правосторонний крипторхизм (правое яичко визуализируется в паховом канале), уменьшение размеров яичек и придатков, объемное образование правого яичка, подозрительное на tumor.

На основании анализа имеющихся лабораторно-инструментальных обследований, а также на основании результатов дообследования, нормального уровня АД, альдостерома была исключена. Повышенный уровень альдостерона на фоне высокого уровня ренина плазмы и нормального АД необходимо интерпретировать как повышенную активность ренин-ангиотензиновой системы с развитием компенсаторного гиперальдостеронизма при декомпенсированной ВДКН.

На основании объективного осмотра, данных инструментальных и лабораторных методов обследования пациенту установлен диагноз:

Основной: «Врожденная дисфункция коры надпочечников. Вирильная форма. Состояние после адреналэктомии слева. Викарная гиперплазия правого надпочечника».

Сопутствующий: «Синдром Клайнфельтера, первичный гипогонадизм, правосторонний крипторхизм. Новообразование правого яичка Эректильная дисфункция. Алиментарно-конституциональное ожирение 1 степени (ИМТ — 30 кг/м²), абдоминальный тип (ОТ — 109 см)».

С учетом этого в отделении пациенту назначена заместительная гормональная терапия препаратом «Кортеф» (гидрокортизон) 30 мг/сутки. Пациент консультирован урологом, рекомендовано определение злокачественного потенциала опухоли, оперативное лечение. Ввиду этого заместительная гормональная терапия препаратами тестостерона на данном этапе не назначена.

## ОБСУЖДЕНИЕ

На примере данного клинического случая мы хотели продемонстрировать сочетание двух эндокринных патологий, для одной из которых характерно повышение уровня андрогенов крови за счет надпочечникового стероидогенеза, а для другой — андрогенный дефицит ввиду сниженной продукции андрогенов в тестикулах. Высокие уровни надпочечниковых андрогенов из-за ВДКН могут уравновешивать частичный дефицит андрогенов тестикулярного происхождения из-за СК. При назначении пациентам с ВДКН заместительной гормональной терапии глюкокортикостероидами гиперсекреция андрогенов в коре надпочечников прекращается, поэтому пациентам необходимо возмещать дефицит тестостерона, вызванный синдромом Клайнфельтера. Одновременное возникновение СК и ВДКН встречается крайне редко. В литературе описаны единичные случаи. Однако есть данные о более высокой частоте одновременного возникновения СК и стероидогенных дефектов. Если рассматривать частоту как ВДКН, так и СК, следует ожидать, что связь между этими двумя заболеваниями встречается чаще, чем описано в современной литературе.

В результате этих особенностей при ведении данного пациента был допущен ряд серьезных ошибок. Пациенту в детстве не проводилось низведение яичка, что увеличило риск развития злокачественного новообразования яичка. Отмена пациентом преднизолона привела к длительной декомпенсации врожденной дисфункции коры надпочечников и развитию гиперплазии коры надпочечников, что неверно расценено как альдостерома и проведена левосторонняя адреналэктомия. Этого состояния можно было бы избежать, если бы пациент получал заместительную гормональную терапию глюкокортикоидами. Отсутствие эффективной стимулирующей терапии препаратом ХГЧ, а затем заместительной гормональной терапии препаратами тестостерона привело к основным симптомам, ассоциированными с дефицитом тестостерона: снижению либидо и эректильной функции, уменьшению безжирового компонента массы тела и мышечной силы, висцеральному ожирению, метаболическому синдрому, мужскому бесплодию, гинекомастии [4].

## ЗАКЛЮЧЕНИЕ

Таким образом, проявление двух заболеваний, при одном из которых наблюдается гиперандрогения, а при другом — андрогенный дефицит у одного пациента обуславливает трудности диагностики и подбора терапии. Ошибки, которые были допущены на различных этапах диагностики и лечения заболеваний у данного пациента, привели к неблагоприятным последствиям. Недостаточная информированность специалистов в этом вопросе приводит к ненужным диагностическим мероприятиям и назначению необоснованного хирургического лечения. Ведение таких пациентов требует мультидисциплинарного подхода, что позволит избежать ошибок и улучшит прогноз и качество жизни данных пациентов.

## ДОПОЛНИТЕЛЬНАЯ ИНФОРМАЦИЯ

Источники финансирования. Работа выполнена по инициативе авторов без привлечения финансирования.

Конфликт интересов. Авторы декларируют отсутствие явных и потенциальных конфликтов интересов, связанных с содержанием настоящей статьи.

Участие авторов. Все авторы одобрили финальную версию статьи перед публикацией, выразили согласие нести ответственность за все аспекты работы, подразумевающую надлежащее изучение и решение вопросов, связанных с точностью или добросовестностью любой части работы.

Согласие пациента. Пациент добровольно подписал информированное согласие на публикацию персональной медицинской информации в обезличенной форме в журнале «Проблемы эндокринологии».
